# Beneficial Effects of *Bacillus amyloliquefaciens* D1 Soy Milk Supplementation on Serum Biochemical Indexes and Intestinal Health of Bearded Chickens

**DOI:** 10.3390/microorganisms11071660

**Published:** 2023-06-26

**Authors:** Liyu Du, Weizhe Chen, Jie Wang, Lingzhu Huang, Qikai Zheng, Junjie Chen, Linhao Wang, Changyu Cai, Xiangbin Zhang, Li Wang, Qingping Zhong, Wujie Zhong, Xiang Fang, Zhenlin Liao

**Affiliations:** 1 College of Food Science, South China Agricultural University, Guangzhou 510642, China; duliyutongxue@163.com (L.D.); superchenweizhe@163.com (W.C.); jiewang@scau.edu.cn (J.W.); huanglingzhu@kingmed.com.cn (L.H.); zqk15897671790@163.com (Q.Z.); wang_lin_hao@163.com (L.W.); caichangyu@stu.scau.edu.cn (C.C.); zhangxb@wens.com.cn (X.Z.); wangli_scau@scau.edu.cn (L.W.); zhongqp@scau.edu.cn (Q.Z.); zwj@scau.edu.cn (W.Z.); 2 College of Animal Science, South China Agricultural University, Guangzhou 510642, China

**Keywords:** *Bacillus amyloliquefaciens*, fermented soy milk, growth performance, serum index, intestinal microbiota

## Abstract

This study investigated the effects of dietary supplementation with *Bacillus amyloliquefaciens* D1 (*B. amyloliquefaciens* D1) on growth performance, serum anti-inflammatory cytokines, and intestinal microbiota composition and diversity in bearded chickens. To investigate the effects of *Bacillus amyloliquefaciensa* and fermented soy milk, 7-day-old broilers were orally fed different doses of *Bacillus amyloliquefaciens* D1 fermented soy milk for 35 days, with the unfermented soy milk group as the Placebo group. This study found that *B. amyloliquefaciens* D1 fermented soy milk improved the intestinal microbiota of broilers, significantly increasing the abundance of beneficial bacteria and decreasing the abundance of harmful bacteria in the gut. *B. amyloliquefaciens* D1 fermented soy milk also significantly reduced the serum lipopolysaccharide (LPS) content. The body weight and daily weight gain of broilers were increased. In conclusion, the results of this study are promising and indicate that supplementing the diets of bearded chickens with *B. amyloliquefaciens* D1 fermented soy milk has many beneficial effects in terms of maintaining intestinal microbiota balance and reducing inflammation in chickens.

## 1. Introduction

The ability of probiotics to encourage growth and prevent infection in animal husbandry has attracted public interest. The direct feeding of microorganisms and probiotics are two viable antibiotic alternatives that feed makers and animal farmers have been actively looking for in recent years. Currently, many studies have shown that *Bacillus* microecological preparations can enhance the immune function of animal organisms. Probiotics can improve broilers’ immune response and protect them from infections, coccidia, viruses, and environmental stresses by altering inflammatory cytokines. Cao et al. [1] found that the addition of *B. amyloliquefaciens* to chicken diets increased the concentration of secreted immunoglobulin A (SIgA) and interleukin 6 (IL-6) in the chicken ileal mucosa significantly and decreased the concentration of tumor necrosis factor-α (TNF-α). When isolated from chicken manure in a study by Lee et al. [2], *B. amyloliquefaciens* KU801 inhibited nitric oxide (NO) production and decreased interleukin-1 alpha (IL-1α) production. In addition, *B. amyloliquefaciens* TL altered the expression of ileal genes, and this *B. amyloliquefaciens* strain reduced the expression of genes related to inflammatory responses, intestinal inflammatory factors, and receptors in broiler ileal mucosa. This suggests that *B. amyloliquefaciens* may reduce inflammation damage to intestinal epithelial cells, thereby reducing pathogen burden and energy expenditure due to immune stimulation and improving broiler growth performance [3]. Moreover, *B. amyloliquefaciens* was found to have the ability to reduce LPS in vitro in a previous study in our laboratory. LPS are often referred to as endotoxins because they cause metabolic endotoxaemia in the circulatory system when they are derived from intestinal microorganisms [4]. According to Ghanima’s research, adding soy milk to broiler feed as a growth promoter improved productivity without having a negative impact on the health of the animals [5]. Soybean is often used as a substrate for fermented foods because of its good nutritional and medicinal properties. Fermented soy milk is an ideal product for developing functional soy foods. Fermentation can effectively improve the digestibility and nutritional functional properties of soy milk, such as high-quality protein, polyunsaturated fatty acids, and several biologically active phenolic compounds, which have health benefits and can improve symptoms of breast cancer, cervical cancer, and menopause in women [6]. A variety of bioactive compounds are produced during the fermentation process of soybean products that are absent from the raw material. Additionally, compared to raw soybeans, fermented soybean products have a higher level of quality and a distinct flavor. During the fermentation process, the microbiota produces protein hydrolases that hydrolyze proteins in soybeans into peptides and free amino acids, which increase the nutritional value and improve the digestive and absorption characteristics of soy foods. In addition, fermentation reduces anti-nutritional components such as protease inhibitors, phytic acid, urease, and oxalic acid [7]. Soy milk is becoming increasingly popular as an alternative to milk, but studies on the effects of soy milk on the intestinal microbiota of animals or humans are scarce.

The chicken intestinal microbiota plays an important role in maintaining intestinal homeostasis and health, mainly through the competitive inhibition of harmful microorganisms and pathogens [8]. Nutrient absorption, feed digestibility, and energy harvest of animals have been reported to be closely related to the intestinal microbiota. Thus, animal productivity is influenced by microbial composition and diversity. Li et al. [9] replaced 25% of soybean meals with fermented soybean meals (FSBMs) to improve growth performance and serum immunity in broiler chickens, which they speculated could be because of the altered microbial composition of the broiler cecum. It has been demonstrated that the supplementation of broiler diets with FSBM increased the number of *Lactobacillus* and reduced the number of *Escherichia coli* (*E. coli*) and *Clostridium perfringens* in the ileum and cecum while improving the growth performance of broilers throughout the growing period. This suggests a synergistic effect between probiotics and fermented feed [10].

In this experiment, bearded chickens were studied by fermenting soy milk with our screened protease-producing *B. amyloliquefaciens* D1 in the basal diet, and unfermented soy milk was the placebo group. We aimed to study the effects on growth performance, serum biochemical parameters, and the intestinal microbiota structure of beard broiler chickens by *B. amyloliquefaciens* D1 to provide a reference for the implementation of of *B. amyloliquefaciens* D1 fermented soy milk in the diets of bearded chickens.

## 2. Materials and Methods

### 2.1. Bearded Chickens and Experimental Design

A total of 42 one-day-old, bearded chickens of similar body weight and normal growth were selected from the Institute of Animal Husbandry, Guangdong Academy of Agricultural Sciences. The 42 bearded chickens were randomly divided into 7 treatment groups, with 6 replicates per group. The first 7 days were the acclimatization period with normal feeding, and the subsequent 7 days were the test period, grouped as shown in Table 1. The same feeding management was adopted, and the experimental period was 35 days long. The chickens were fed and watered freely, and 24 h of light was maintained daily (24 h of fluorescent light). At 7 days of age, the chickens were vaccinated with Newcastle disease via nasal drops and eye drops. At 14 days of age, the chickens were vaccinated with Fasciola triple vaccine via nasal drops. At 21 days of age, the chickens were vaccinated again with Newcastle disease via eye drops (all vaccines were obtained from Ha Pharmaceutical Group Biological Vaccine Co., Harbin, China). Before the experimental grouping, the chickens were starved for 12 h to empty their intestines. During the experiment, the amount of feed delivered and remaining feed in each column was recorded daily, the number of chickens surviving in each column was recorded, and the average daily feed consumption per column was calculated. The feed was withdrawn 12 h before the end of each phase of the experiment to allow the chickens to keep their fasting, after which the weight of the chickens in each cage was weighed and the average weight of the chickens in each pen was calculated. Finally, the average daily gain (ADG), average daily feed intake (ADFI), and average feed/Gain ratio (F/G) were calculated for each pen at each growth stage.

### 2.2. Preparation of Soy Milk

Pure soy milk powder (5 g) was mixed with distilled water (50 mL) in a 250 mL conical flask. After inoculation with 10^9^ cfu/g of D1, the soy milk was fermented at 37 °C and 180 rpm for 72 h.

### 2.3. Calculation of Production Performance Indexes

The average daily feed intake (ADFI), average daily gain (ADG), and average feed/gain ratio (F/G) were calculated for each replicate group.

### 2.4. Sample Collection and Treatment

After 35 d of experimental intervention, three chickens were randomly selected from each replicate. Blood samples were collected from wing veins, and then the birds were sacrificed by cervical dislocation. Cecal contents and cloacal samples were aseptically collected from each bird. Blood samples were allowed to clot at room temperature before centrifugation at 4000× *g* for 10 min for serum collection. Samples were immediately placed in an ice box and then stored at −80 °C for subsequent index measurements. Blood serum samples were assessed for 9 blood biochemistry parameters at the Guangdong Animal Experiment Center (Guangzhou, China), and the cecum contents were sent to Shanghai Ling’en Biotechnology Co., Ltd. (Shanghai, China) for PacBio sequencing.

### 2.5. Statistical Analysis

*p* values calculated using an ordinary one-way ANOVA with Tukey’s multiple comparisons test. Analysis was performed using GraphPad Prism software version 7.0.

## 3. Results

### 3.1. Effect of B. amyloliquefaciens D1 Fermented Soy Milk on the Productive Growth Performance and Serum Biochemical Indexes of Bearded Chickens

The daily weight gain of the broilers in the *B. amyloliquefaciens* D1 fermented soy milk group was higher than that of the soy milk control group after 35 days of the trial (Table 2). The differences in feed intake and weight gain ratio were not significant between 7 and 42 days of age, but the *B. amyloliquefaciens* D1 fermented group showed a gradual decrease in weight gain ratio with the increase in the amount of fermented soy milk added. Broilers fed diets supplemented with *B. amyloliquefaciens* D1 fermented soy milk showed greater weight gain from 7 to 42 days compared to those on diets without *B. amyloliquefaciens* D1. The results showed a beneficial trend, but one of the limitations of the present study was the relatively small number of replicates per group. The effect of the relatively small number of replicates can be observed in the statistical results of the data.

The effects of dietary supplementation of *B. amyloliquefaciens* D1 fermented soy milk on the serum physiological and biochemical indicators of broiler chickens are shown in Table 3. The table shows that adding *B. amyloliquefaciens* D1 fermented soy milk to the chickens’ diets had no significant effect (*p* > 0.05) on the chickens’ serum Triglyceride (TG) and Total protein (TP) content. The difference in the Albumin/Globulin (A/G) between the groups was insignificant (*p* > 0.05), indicating that the application of *B. amyloliquefaciens* D1 did not cause the broilers to be hyperimmune. The fermented soy milk with *B. amyloliquefaciens* D1 at 50% and 100% feedings had higher TP and Albumin (ALB), as well as increased A/G compared to unfermented soy milk at the same feedings. The differences in serum Aspartate aminotransferase (AST), Alanine aminotransferase (ALT), and ALT/AST among the test groups were not significant (*p* > 0.05), indicating that the application of *B. amyloliquefaciens* D1 had no adverse effects on the liver function of the broilers.

**Figure 1:**
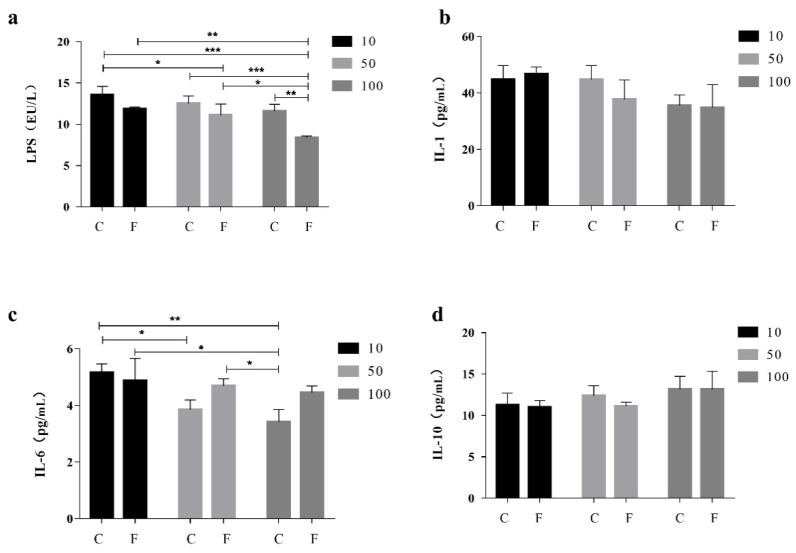
Effect of fermented soy milk on serum LPS, IL-1, IL-6, and IL-10 indexes in broiler chickens. C control group; F fermentation solution group; numbers are percentages of soy milk addition; CK is the normal diet group (Mean ± SD). * *p* < 0.05, ** *p* < 0.001; *** *p* < 0.001. (**a**) Levels of LPS in broiler serum, (**b**) Levels of IL-1 in broiler serum, (**c**) Levels of IL-6 in broiler serum, (**d**) Levels of IL-10 in broiler serum.

Compared to the CK, The level of LPS in the serum of the F100 group was significantly lower than that of the CK by 37.68% (Figure 1a), and it was lower than that of the C100 and C10 groups by 27.80 and 38.25 percent, respectively. Interleukin-1 (IL-1) and Interleukin-10 (IL-10) levels did not differ significantly (Figure 1b,d), whereas serum IL-1 levels in the F100 group were 22.51% lower than those in the F10 group. Increased levels of IL-6 were found in the groups treated with F-10, F-50, and F-100 (*p* < 0.05) (Figure 1c), with a 22.98% increase in serum Interleukin-6 (IL-6) levels in the F100 group compared to the C100 group and an 8.68% decrease in serum IL-6 levels in the F100 group compared to the F10 group.

### 3.2. Effect of alpha Diversity Index of Fermented Soy Milk Broiler Cecum Contents by B. amyloliquefaciens D1

An *alpha* diversity analysis was performed to evaluate the overall differences in the structure of the cecum microbial community between the fermented and unfermented soy milk groups and the blank control group. As shown in Table 4, the Chao1 and ACE indices measured species abundance, and the Richness and Shannon indices were used to measure species diversity. The results showed that the differences in ACE and Chao1 indexes among broiler groups were not significant (*p* > 0.05), indicating that the addition of soy milk and fermented soy milk did not have significant effects on the abundance of cecum microbiota in broilers. The changes in species variety across the seven experimental groups were not statistically significant, according to the Shannon index and Simpson index (*p* > 0.05). This indicates that there was no difference in species richness and diversity of cecum microbiota between the groups.

### 3.3. Effect of B. amyloliquefaciens D1 Fermentation of Soy Milk Broiler Cecum Contents on β-Diversity of Bacterial Microbiota

**Figure 2:**
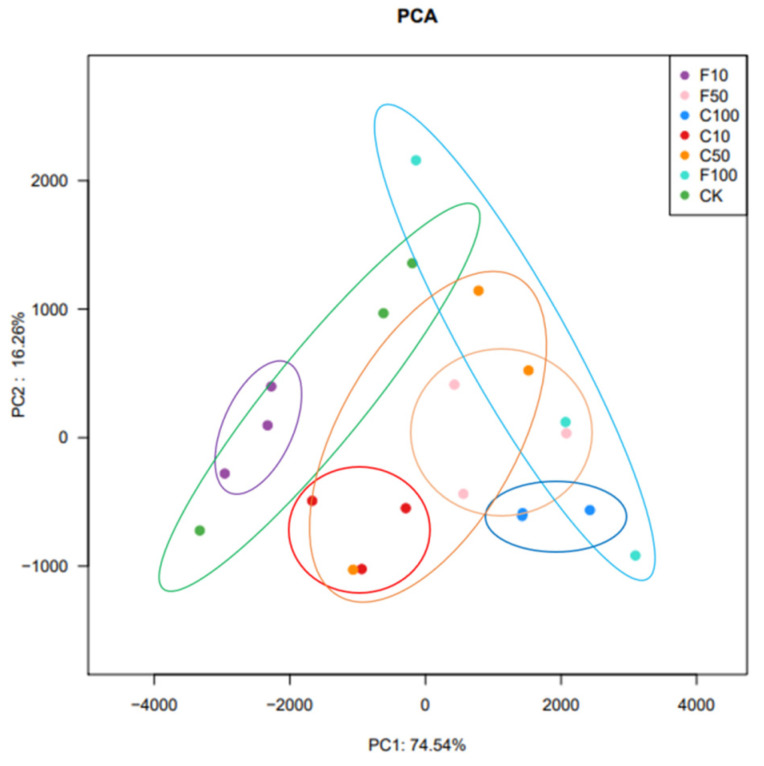
Control (CK, green), unfermented (C10, C50, C100), and fermented soy milk (F10, F50, F100) groups with OTU showed significant clustering at the 97% identity level.

The *β* diversity analysis in this experiment was performed based on the unweighted Unifrac distance matrix of the seven communities and the differences between sample groups were further assessed using principal component analysis (PCA), as shown in Figure 2. The results showed that there were some differences in microbial composition among the seven groups, with the presence of especially the F10 and F100 groups. The results showed that the microbial composition of the broiler cecum changed with increasing *B. amyloliquefaciens* D1. there was some distance between groups C and F, indicating that feeding *B. amyloliquefaciens* D1 fermented soy milk affected the microbial composition of the broiler cecum microbiota.

**Figure 3:**
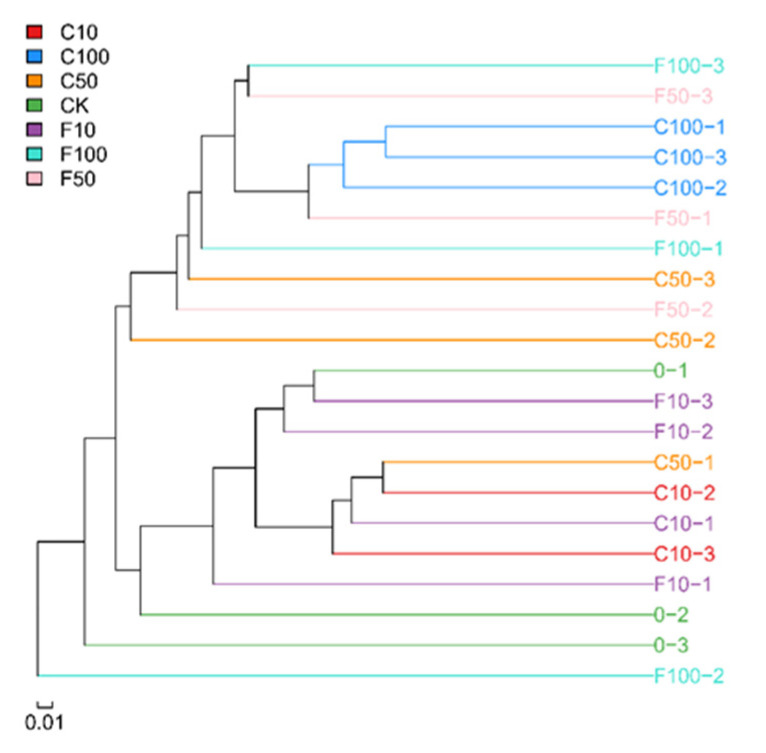
Dendrogram of the similarity of the samples.

Figure 3 displays the outcomes of the unweighted pair-group method with arithmetic means-based inter-sample cluster analysis. Broiler cecum microbes were grouped into three groups, demonstrating that there were some variations between the microbes in fermented soy milk made with *B. amyloliquefaciens* D1 and those in unfermented soy milk. The C10 group and C100 group both formed one cluster, indicating that the unfermented group had a high degree of commonality. Due to the interleaved additions and the more complicated effects of the *B. amyloliquefaciens* D1 fermented soy milk, the three parallels of the other groups did not cluster together.

### 3.4. Effect of B. amyloliquefaciens D1 Fermentation of Soy Milk on the Structural Composition of Microbiota in Broiler Cecum Contents

**Figure 4:**
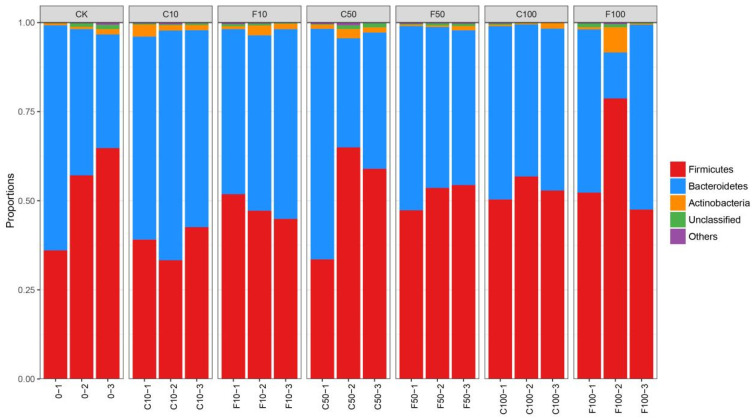
Microbiota composition of broiler cecum at the phylum level. It was divided into the following seven groups: diet group (CK), unfermented group (C10, C50, C100), and *B. amyloliquefaciens* D1 fermented soy milk group (F10, F50, F100).

Figure 4 shows that the three most prevalent microorganisms in terms of relative abundance in the broiler cecum microbiota level were dominated by Firmicutes, followed by Bacteroidetes, and then Actinobacteria. The ratios of Firmicutes and Bacteroidetes were relatively stable. The overall trend in the abundance of genera in the broiler cecum was an increase in the F/B with increasing addition in the *B. amyloliquefaciens* D1 fermented group compared to the unfermented soy milk group (Figure 4).

**Figure 5:**
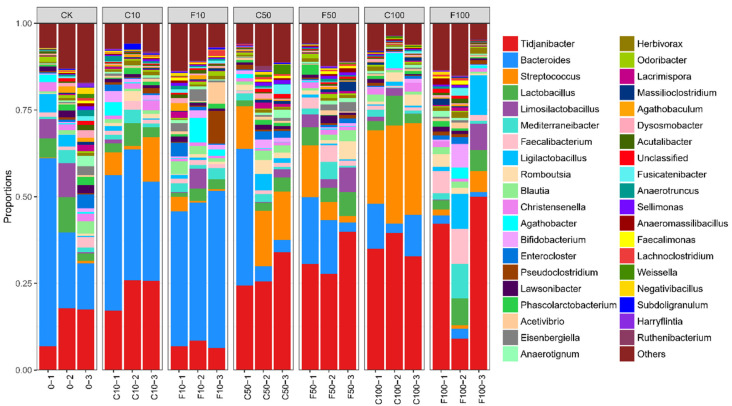
Microbiota composition of the cecum of broiler chickens at the genus level. It was divided into the following seven groups of three parallels each: diet group (CK), unfermented group (C10, C50, C100), *B. amyloliquefaciens* D1 fermented soy milk group (F10, F50, F100).

Figure 5 shows the top 10 microorganisms in terms of relative abundance at the level of the microbial genus in the broiler cecum: *Tidjanibacter*, *Bacteroides*, *Streptococcus*, *Lactobacillus*, *Limosilactobacillus*, *Mediterraneibacter*, *Faecalibacterium*, *Ligilactobacillus*, *Romboutsia*, *Blautia*.

**Figure 6:**
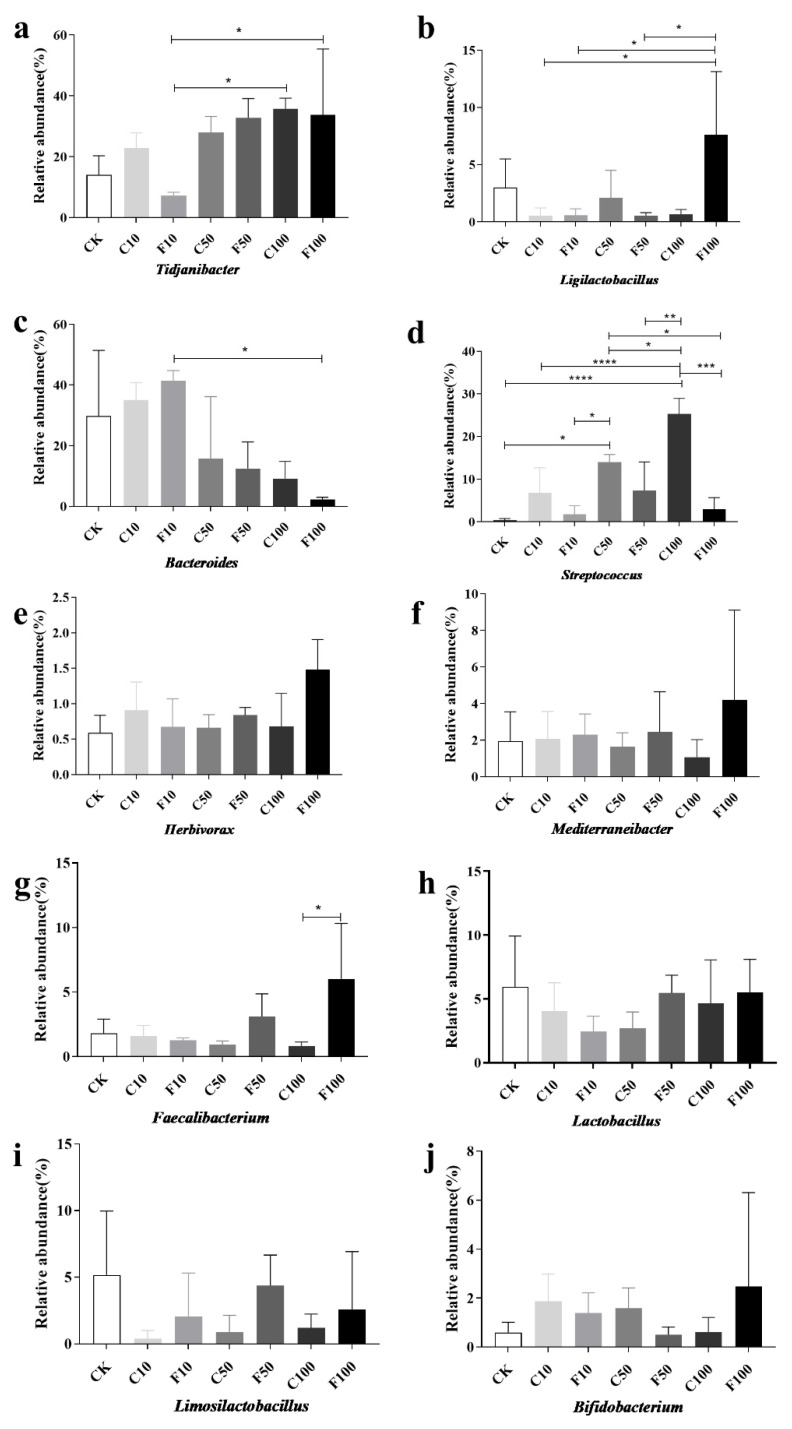
Abundance of ten genus microorganisms with greater than 1% abundance in the cecum of broiler chickens at the genus level among the different groups (**a**–**j**). They were divided into the following seven groups: diet group (CK), unfermented group (C10, C50, C100), and *B. amyloliquefaciens* D1 fermented soy milk group (F10, F50, F100) (Mean ± SD). * *p* < 0.05, ** *p* < 0.001; *** *p* < 0.001, **** *p* < 0.0001.

Figure 6 displays the strains chosen for investigation with a relative abundance of 1% or more and with notable and specific variation patterns. At the genus level (Figure 6), the most representative genus in CK included *Bacteroides* (29.76%), *Tidjanibacter* (14.05%), *Lactobacillus* (5.95%), *Limosilactobacillus* (5.17%), and *Ligilactobacillus* (3%), while the most representative genus in the F100 group included *Tidjanibacte* (33.75%), *Ligilactobacillus* (7.61%), *Faecalibacterium*(6.03%), *Lactobacillus* (5.50%), and *Mediterraneibacter* (4.19%). The relative abundance of other items was less than 3%. 

As shown in Figure 6, the genus with significant differences in relative abundance included *Tidjanibacter*, *Ligilactobacillus*, *Bacteroides*, *Streptococcus*, and *Faecalibacterium*. Compared with the F10 group, the relative abundance of *Tidjanibacter* and *Ligilactobacillus* in the F100 group increased significantly (*p* < 0.05) (Figure 6a,b), while the relative abundance of *Bacteroides* decreased significantly (*p* < 0.05) (Figure 6c). It is possible that *B. amyloliquefaciens* D1 fermented soy milk regulated the cecum microbiota of broiler chickens, resulting in an increase in beneficial intestinal bacteria and a decrease in harmful ones. Similar trends were observed for *Ligilactobacillus*, *Faecalibacterium*, *Herbivorax*, *Mediterraneibacter*, and *Bifidobacterium* (Figure 6b,e–g,j). All were in the highest abundance in the F100 group, probably due to the regulatory effect of *B. amyloliquefaciens* D1. A significant increase in the abundance of *Streptococcus* occurred with increasing soy milk addition, but the abundance of *B. amyloliquefaciens* D1 fermented soy milk group decreased compared to unfermented soy milk in each gradient, mainly due to a significant decrease in *Streptococcus alactolyticus* and *Streptococcus gallolyticus* were significantly reduced (Figure 6d), which indicates that *B. amyloliquefaciens* contributed to reducing harmful bacteria. 

**Figure 7:**
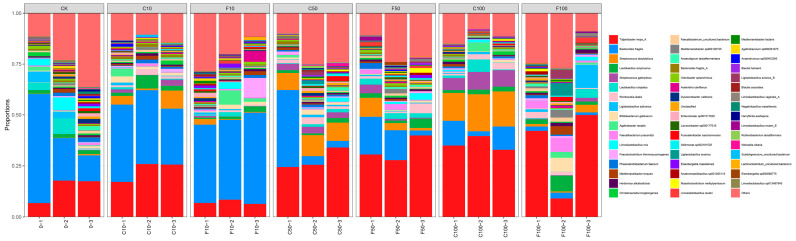
Microbiota composition of broiler cecum at the level of species. It was divided into the following seven groups of three parallels each: diet group (CK), unfermented group (C10, C50, C100), *B. amyloliquefaciens* D1 fermented soy milk group (F10, F50, F100).

Strains was a relative abundance of 1% or more were selected for analysis, and those with significance and some variation patterns are shown in Figure 7. At the species level, the most representative species in F100 included *Tidjanibacter inops_A* (33.75%), *Lactobacillus salivarius* (4.23%), *Faecalibacterium prausnitzii* (3.95%), *Lactobacillus amylovorus* (3.89%), and the relative abundance of other species was less than 3%. The most representative species in the C100 group included *Tidjanibacter inops A* (35.82%), *Streptococcus alactolyticus* (16.35%), *Bacteroides fragilis* (8.65%), and *Streptococcus gallolyticus* (7.59%).

**Figure 8:**
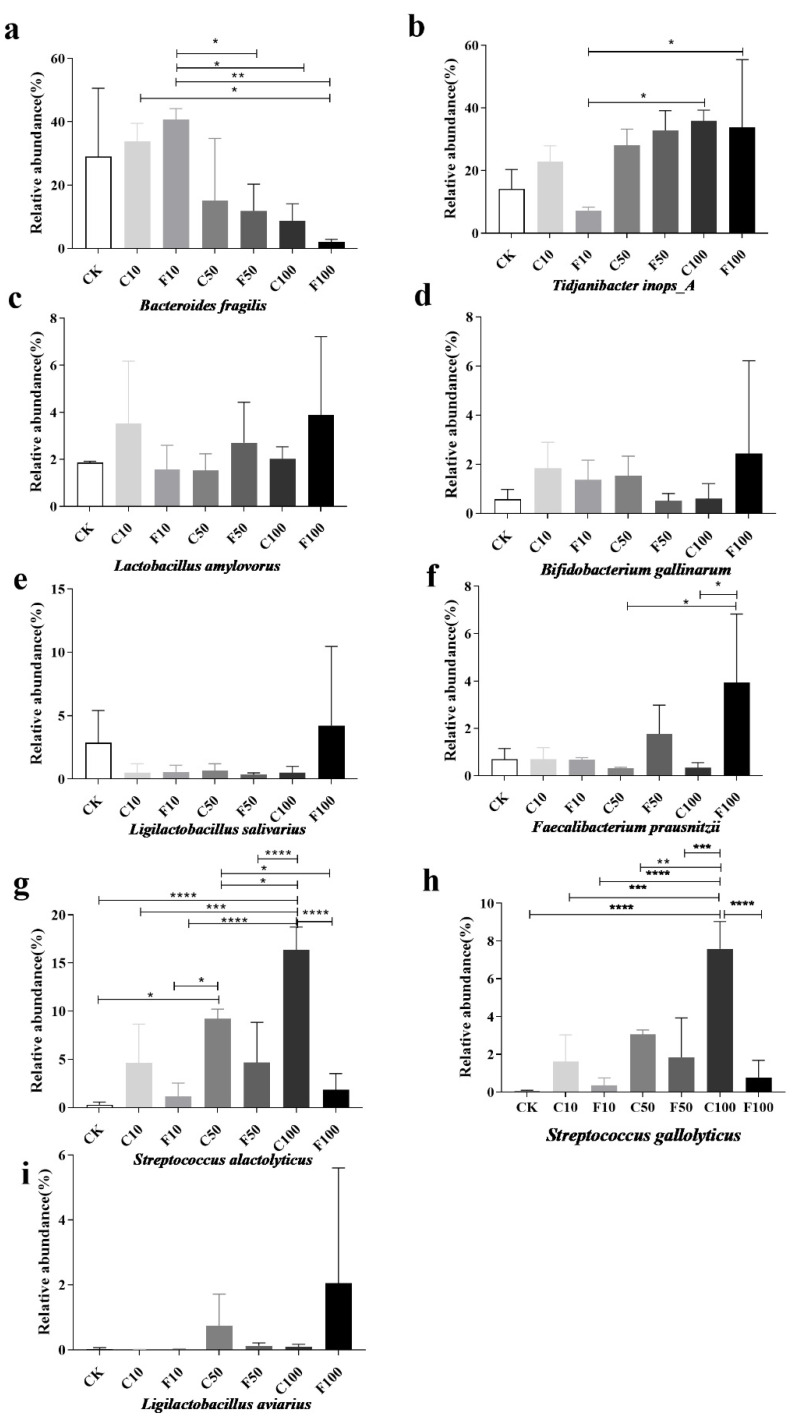
Abundance of nine species of microorganisms with greater than 1% abundance in the broiler cecum at the level of species among different groups (**a**–**i**). They were divided into the following seven groups: diet group (CK), unfermented group (C10, C50, C100), *B. amyloliquefaciens* D1 fermented soy milk group (F10, F50, F100) (Mean ± SD). * *p* < 0.05, ** *p* < 0.001; *** *p* < 0.001, **** *p* < 0.0001.

The increased relative abundance of *Streptococcus alactolyticus* and *Streptococcus gallolyticus* may play a pathogenic role in the growth of broilers, while the abundance of probiotic bacteria in the cecum microbiota of broilers was increased in the F100 group due to fermentation and the effect of *B. amyloliquefaciens*. The abundance of *B. fragilis* was significantly lower in the F100 group compared to the F10 group (*p* < 0.05) (Figure 8a). *Tidjanibacter inops_A* gradually increased with the addition of soy milk, *Lactobacillus amylovorus Bifidobacterium gallinarum*, *Lactobacillus salivarius*, and *Ligilactobacillus aviaries* had the highest abundance in the F100 group but there was no significant difference between the groups (*p* > 0.05) (Figure 8b–e,i).

*Streptococcus alactolyticus* was more prevalent in the unfermented group than in the same amount of the *B. amyloliquefaciens* D1 fermented group with the increase in soy milk addition, and it peaked at the C100 group with the increase in *B. amyloliquefaciens* D1 fermented soy milk addition (Figure 8g). The cecum microbiota was modified by *B. amyloliquefaciens* D1 in response to the addition of more fermented soy milk. This result showed that the relative abundance of *Streptococcus alactolyticus* increased significantly after the addition of unfermented soy milk. The abundance of *Streptococcus gallolyticus* in the unfermented soy milk was greater than fermented soy milk (Figure 8h). 

### 3.5. Correlation Analysis of Strains of Broiler Intestinal Microbiota Species Level with Immune Factors and ADG

**Figure 9:**
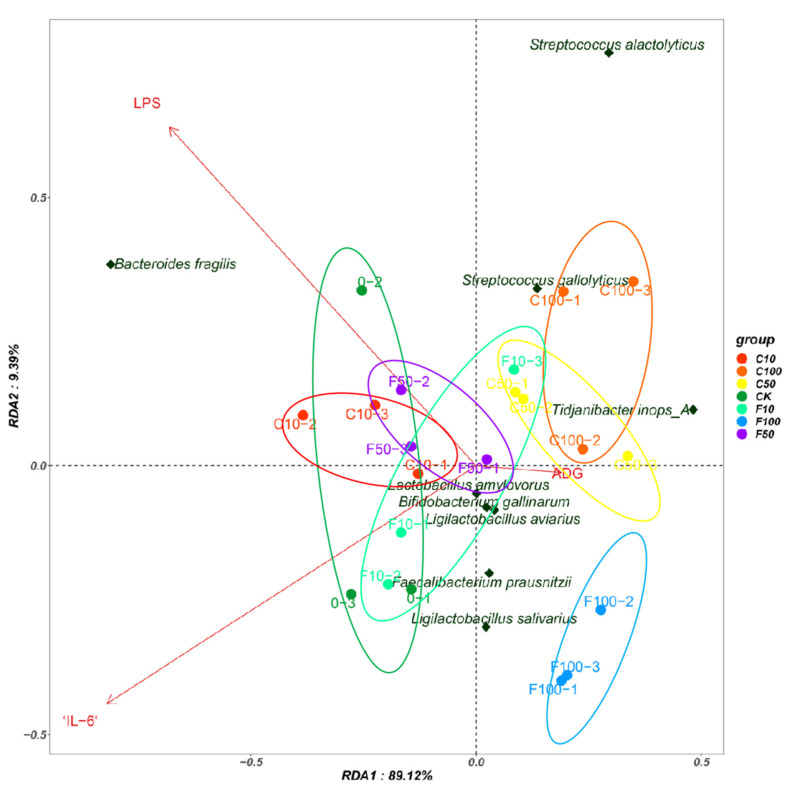
Redundancy analysis between the broiler intestinal microbiota species level, immune factors, and ADG.

Redundancy analysis (RDA) was used to explain possible correlations between the cecum bacterial community and the inflammatory factors IL-6, LPS, and the average daily weight gain (ADG) index in broiler chickens. RDA showed cumulative contributions of the factors to the cecum intestinal microbiota of 89.12% and 9.39%, respectively (Figure 9). At the species level, the results showed a positive correlation between *Bacteroides fragilis* and LPS, a positive correlation between *Tidjanibacte inops A* and ADG, and a negative correlation with IL-6.

**Figure 10:**
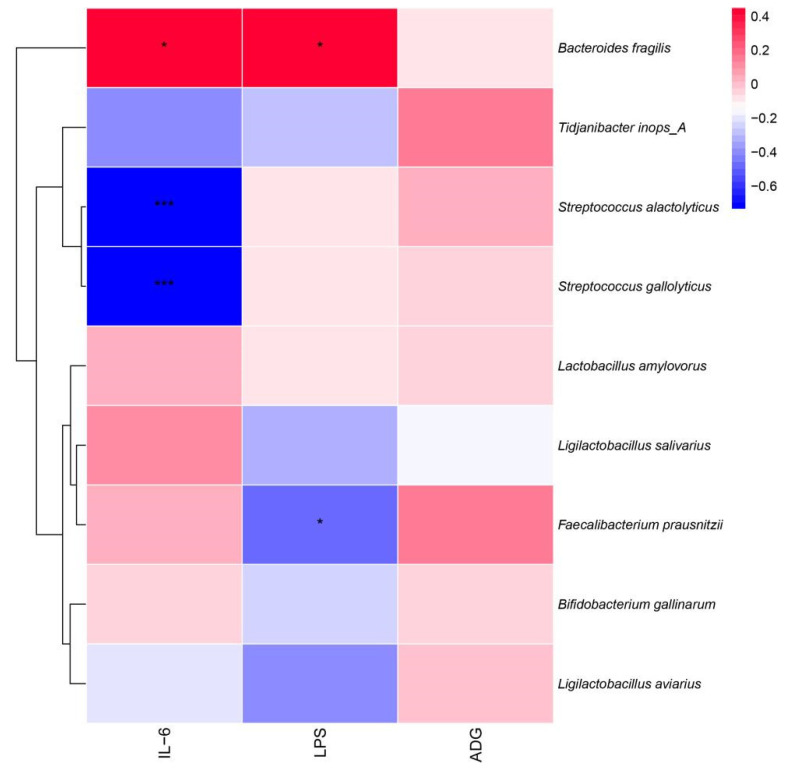
Pearson analysis between the broiler intestinal microbiota species level, immune factors, and ADG. * *p* < 0.05, *** *p* < 0.001.

Pearson correlation analysis was used to correlate the intestinal microbiota with inflammatory factors and ADG (Figure 10). The results showed that *Faecalibacterium prausnitzii* showed a significant negative correlation with LPS (*p* < 0.05) and a significant positive correlation between *Bacteroides fragilis* and IL-6 and LPS (*p* < 0.05), while *Streptococcus alactolyticus* and *Streptococcus gallate* both showed a significant negative correlation (*p* < 0.05) with IL-6.

## 4. Discussion

To investigate the in vitro probiotic effect of *B. amyloliquefaciens* D1 and fermented soy milk together, a previously experimentally screened strain of *B. amyloliquefaciens* D1 with high protease production was used directly in this study to ferment soy milk and implement it into the normal diet of bearded chickens, with unfermented soy milk acting as the control. It was shown that soy milk supplemented with *B. amyloliquefaciens* D1 increased the body weight of broilers at 35 days and reduced the F/G ratio. The results indicated that *B. amyloliquefaciens* D1 as a probiotic could promote growth and improve feed efficiency. We chose to focus our investigation on bearded chickens because they are a prominent local breed of chicken that is exclusive to China and they have a distinctive meat quality [11]. In addition, the bearded chicken has a high economic and research value. Consumers have recently preferred slow-growing broilers, such as bearded chickens [12]. 

Bai et al. showed that although the addition of the probiotic *Bacillus subtilis* (*B. subtilis*) to the diet did not have any effect on the growth performance of broilers from 1 to 21 days, broilers fed the probiotic showed higher ADG and ADFI and lower F/G ratio compared to broilers who were not fed the probiotic [13]. In a study by Ahmat et al. [14], it was observed that broilers fed *B. amyloliquefaciens* LFB112 or its metabolites had higher body weights than the control and antibiotic groups, especially at day 39, with a significant increase in daily weight gain and a decrease in ADFI. Sun et al. used *B. subtilis* BJ-1 to ferment cottonseed meals and added 40 and 80 g/kg of fermented cottonseed to the diet, increasing the body weight of broilers at the start and throughout the feeding period [15]. While performance improvements in these experiments varied, it was clear that probiotic supplementation could improve broiler chickens’ productivity.

Serological parameters are indicators of the health status of broiler chickens and are influenced by the type of feed and its nutritional composition. Serum biochemical parameters are indicators of the physiological, nutritional, and pathological status of broilers and can be correlated to determine the influence of nutritional factors and additives in the ratio [16]. TP is an important indicator of protein deposition in animals. Albumin is mainly synthesized by the liver and is responsible for transporting metabolites in the body, maintaining colloidal osmotic stability, and protecting globulins in the blood. Serum albumin and globulin are the two major components of total protein. ALB/GLB reflects the immune status of the body; a lower ratio of ALB/GLB indicates that the body synthesizes more globulin for improving the immune function of the body and is also an important indicator of liver injury and function [17]. However, in this experiment, except for the low dose group (10% soy milk addition), the total protein and A/G of the *B. amyloliquefaciens* D1 fermented group were higher than those of the unfermented soy milk group, within which it was controlled, and the serum protein indexes of the *B. amyloliquefaciens* D1 group were similar to those of the CK group. We speculate that the reason for these results is that soy milk contains anti-nutritional factors that interfere with the absorption of nutrients in broilers, and *B. amyloliquefaciens* D1 fermented soy milk reduces the content of anti-nutritional factors; therefore, the total serum protein indexes increased compared to the unfermented group, while the CK group was fed only scientifically formulated diets, so there was no significant difference in the total serum protein and white globule ratio between the *B. amyloliquefaciens* D1 fermented soy milk group and the CK group. There were no significant differences between the *B. amyloliquefaciens* D1 fermented soy milk and CK groups. There was no discernible differences between the *B. amyloliquefaciens* D1 fermented soy milk group and the CK group in terms of total serum protein and the A/G since the CK group received only scientifically designed diets. 

The disruption of the structural and functional integrity of the hepatocyte membrane leads to increased ALT and AST activity. These soluble enzymes then enter the bloodstream from the hepatocytes, leading to increased serum enzyme activity. Therefore, serum ALT and AST activity is a specific indicator of the degree of hepatocyte damage [18]. The results of this experiment showed that the application of *B. amyloliquefaciens* D1 fermented soy milk did not affect broiler serum ALT, AST, and ALT/AST, indicating that the addition of *B. amyloliquefaciens* D1 fermented soy milk to the diet did not adversely affect the growth and development of broiler liver.

Concentrations of lipid components such as chicken serum triglycerides were used to measure lipid metabolism. No regular and significant differences were observed in the TG content of broilers fed with *B. amyloliquefaciens* D1 fermented soy milk, indicating that *B. amyloliquefaciens* D1 fermented soy milk had little effect on TG content. The results of our experiment were similar to those of a study by Saleh et al. [17], in which broiler chickens fed wet feed fermented with *Bacillus licheniformis* were found to have no effect on TG and total cholesterol concentrations. We examined the gene expression levels of pro-inflammatory IL-1 and IL-6 and anti-inflammatory IL-10 in broiler serum as well as LPS levels. In Figure 1, IL-1 levels were slightly decreased in the group-fed soy milk as the proportion of soy milk increased, while the decreasing trend was greater in the group-fed fermented soy milk with *B. amyloliquefaciens* D1 compared to unfermented soy milk, indicating that soy milk can reduce inflammation. The combined effect of fermented soy milk and *B. amyloliquefaciens* D1 can also reduce IL-1 levels. There was an overall trend of decreasing IL-6 production, but fermented soy milk containing *B. amyloliquefaciens* D1 had a significant increase in IL-6 production relative to the unfermented soy milk group. The *Akkermansia muciniphila* (*A. muciniphila*) has an important role in maintaining the body’s immune system and delaying the functional aging of the organism. It was found that the active compound (a15:0-i15:0 PE), a major component of the lipid membrane of *A. muciniphila*, promoted the release of IL-6 to regulate the host immune response [19]. It can be hypothesized that *B. amyloliquefaciens* can produce amylase, protease, or parts of the bacterium itself in the broiler intestine that can induce the chicken immune system and increase the secretion of IL-6 and IL-10. *B. amyloliquefaciens* D1 may also be associated with a mechanism similar to that of *A. muciniphila* in regulating immunity. In a study by Mazanko et al., the production of IL-6 and IL-10 in broiler serum was significantly higher when fed Bacillus probiotics, and the production of IL-6 was higher than that of IL-10 [20]. Yitbarek et al. showed a similar increase in IL-6 and IL-10 secretion when broilers were fed with a mixed probiotic strain and concluded that the increase in pro-inflammatory and anti-inflammatory cytokines indicated beneficial immunomodulation and the maintenance of immune homeostasis [21]. Gadde et al. observed an elevated secretion of IL-6 in the ileum of the *B. subtilis* group compared to the control group, and a significant increase in occludin was also observed, leading to the conclusion that *B*. *subtilis* affects intestinal barrier integrity and alters intestinal immune activity by increasing the expression of tight junction genes [22]. Similar conclusions were obtained by Guo et al. in a study on the immune system and growth performance of chickens, where *B. subtilis* prevented bacterial diseases and maintained the integrity of the intestinal epithelium by activating NF-κB [23]. We speculate that the elevated secretion of both IL-6 and IL-10 in broiler serum is associated with the maintenance of intestinal barrier integrity. In our previous laboratory study, the in vitro LPS degradation rate of Trypticase Soy Broth of *B. amyloliquefaciens* D1 exceeded 97% in 4 h. In our in vivo broiler chickens experiments, the degradation rate of LPS in the F100 group was 27.8% compared to the C100 group and 37.7% compared to the CK group. We speculate that the in vivo reduction in LPS degradation rate by D1 may be due to the interaction of complex factors in vivo. This demonstrates that *B. amyloliquefaciens* D1 can degrade LPS both in vivo and in vitro. The reduction in serum LPS levels is closely related to intestinal microbiota. A balanced intestinal microbiota reduces the entry of LPS into the serum, which in turn reduces the production of inflammation in the broiler organism [4]. This study demonstrated that *B. amyloliquefaciens* had a protective effect on LPS-induced intestinal mucosal damage and that dietary supplementation with *B. amyloliquefaciens* could effectively improve the intestinal antioxidant and immune status of broiler chickens in the early stages [24].

The bacteria in the intestinal tract are large and complex micro-ecosystems that have co-evolved with the host and play an important role by directly participating or indirectly influencing nutrient digestion and absorption, energy supply, lipid metabolism, and immune regulation of the organism. Changes in the microbiota can profoundly affect the host’s internal homeostasis. In chicken production, bacteria associated with productivity mainly include *Firmicutes*, *Bacteroidetes*, and *Proteobacteria* [25]. The F/B ratio is considered an indicator of obesity due to the increased energy harvesting capacity of *Firmicutes* [26], but carbohydrate fermentation by *Firmicutes* produces large amounts of short-chain fatty acids that are both immunomodulators and energy sources [27]. While there was no significance among the groups in this study, the F/B ratio tended to increase in the *B. amyloliquefaciens* D1 fermentation group, and we speculate that due to higher F/B in the cecum microbiota the efficiency of feed energy utilization improves, which may contribute to the increased body weight of broilers. It was shown that the addition of more dietary fiber could increase the amount of *Bacteroides* and decrease the ratio of F/B [28]. Soy milk is rich in dietary fiber, and the breakdown of dietary fiber in fermented soy milk by the action of *B. amyloliquefaciens* resulted in a higher amount of *Bacteroides* in the unfermented soy milk group, although these speculations require further study. In our study, at the genus level, the relative abundance of *Tidjanibacte*, *Lactobacillus, Faecalibacterium*, *Mediterraneibacter*, *Herbivorax*, and *Ligilactobacillus* was upregulated in the *B. amyloliquefaciens* D1 fermentation group, decreasing the abundance of two genus (*Bacteroides* and *Streptococcus*). The available findings suggest that Poria brick tea’s main purified fraction and the extracellular polysaccharide EPSs-2 of the *Aspergillus Christi* can not only reduce intestinal cell damage but also promote the production of short-chain fatty acids and modulate microbial composition by increasing the growth of *Tidjanibacter* [29]. However, due to the lack of studies on *Tidjanibacter*, its ecological role and pathogenic potential are not known. In our experimental results in broiler cecum, *Faecalibacterium* in the main species is *Faecalibacterium prausnitzii*, which has the potential to prevent inflammation and is the most important butyrate-producing bacterium in the human colon. Butyrate is a key colon health regulator as it protects the colon from inflammation and colorectal cancer [30]. Many studies have demonstrated that *Faecalibacterium* deficiency is associated with microbial dysbiosis, which may cause inflammation and, in severe cases, chronic diseases such as psoriasis, hypertension, and heart and kidney disease [31]. *Ligilactobacillus aviarius* and *Lactobacillus amylovorus* were enhanced in the fermentation group. The increased abundance of *Lactobacillus* in the gastrointestinal tract of chickens is thought to be beneficial for their health and performance. *Ligilactobacillus aviarius* is a promising probiotic with multiple applications in human and animal health, especially in the regulation of the intestinal microbiota and immune system and in broiler chickens for the prevention of Salmonella infections [32]. Chen et al. also found that *Ligilactobacillus aviarius* had anti-pig epidemic diarrhea virus activity. Through the microorganisms themselves and their metabolites, particularly lactic acid bacteria, microorganisms can control the gut microbiota and intestinal mucosal immune system [33]. *Lactobacillus amylovorus* is a lactic acid bacterium that efficiently utilizes starch and has shown excellent probiotic effects such as increasing daily weight gain and resistance in animal production [34,35]. Shen et al. studied the enzymatic profile of *Lactobacillus amylovorus* SLZX20-1 and found that it can produce many enzymes [36], for example, aminopeptidase and active α-galactosidase. α-Galactosidase can hydrolyze α-galactoside, a common antinutritional factor in legumes, which affects the absorption of host nutrients [37]. The increased abundance of *Lactobacillus amylovorus* in the cecum of broiler chickens supplemented with soy milk better promotes the absorption of nutrients in broilers. *B. amyloliquefaciens* D1 could promote the growth of beneficial *Lactobacillus*. The abundance of *Bacteroides fragile* decreased with the increasing soy milk supplementation in a dose-dependent manner. Similarly, An et al. treated ducks with *Lactobacillus Plantarum* at 400 and 800 mg/kg, respectively. Both increased the relative abundance of *Firmicutes* and decreased *Bacillus fragilis*, with a parallel decrease in the relative abundance of *Bacillus fragilis* occurring in the cecum [38]. Similarly, Wang et al. treated broilers with both screened probiotics to significantly reduce the relative abundance of zoonotic enteric pathogens (*E. coli* and *Bacteroides fragilis*), suggesting a potential probiotic role in the inhibition of broiler enteric pathogen colonization and the pathogen contamination of poultry products by this probiotic [39]. 

We were able to identify that *Streptococcus alactolyticus* and *Streptococcus gallolyticus* were significantly reduced in the *B. amyloliquefaciens* D1 fermented soy milk group. *Streptococcus* colonized in humans and animals may be opportunistic pathogens that induce a variety of diseases and inflammatory conditions. Moreover, the abundance of *Streptococcus gallolyticus* in the C100 group accounted for 7.59%, which was the highest abundance of *Streptococcus gallolyticus* among all groups. *Streptococcus gallolyticus* is commonly found in the intestines of koalas, birds, ruminants, and humans, etc. *Streptococcus gallolyticus* plays a crucial role in herbivore digestion through its ability to break down plant sugars and has a specific ability to degrade tannins and other toxic metabolites such as gallate [40]. The presence of anti-nutritional factors such as tannins and phytic acid in soybean may cause the proliferation of *Streptococcus gallolyticus*; therefore, the abundance of *Streptococcus gallolyticus* was highest at the maximum amount of soy milk addition. Fermentation may have reduced the anti-nutritional factors in soy milk [41], meaning that the abundance of *Streptococcus gallolyticus* was reduced. However, *Streptococcus gallolyticus* causes infectious endocarditis in chickens, and healthy chickens may possess the bacterium in their normal flora as an opportunistic pathogen [42]. *Streptococcus gallolyticus* may be part of the gut microbiota of clinically healthy broilers, but they also represent a high hygienic risk. Mokrani et al. found that potentially pathogenic *Streptococcus alactolyticus* were greatly increased in obese rats and sharply decreased with grape seed and peel extract treatment and other potentially pathological strains [43]. We hypothesized that the addition of *B. amyloliquefaciens* D1 fermented soy milk could improve production performance by positively regulating the abundance of beneficial intestinal bacteria and reducing the abundance of harmful bacteria. 

According to the results of the RAD and Pearson correlation analysis, dangerous bacteria like *B. fragilis* were shown to have a positive association with the pro-inflammatory components IL-6 and LPS, while *Tidjanibacte inops A* had a negative correlation with IL-6. We hypothesize that fermented soy milk may boost broiler immunity by enhancing the intestinal microbiota because *Tidjanibacte inops A* abundance was much greater and *B. fragilis* abundance was significantly lower in the high-dose fermented soy milk group. *Faecalibacterium prausnitzii* abundance was significantly increased in the fermented soy milk group, while LPS levels were significantly negatively correlated with *Faecalibacterium prausnitzii* abundance. D1 may have reduced serum LPS levels in the organism by increasing the abundance of beneficial bacteria. *Streptococcus alactolyticus* and *Streptococcus gallolyticus* were negatively correlated with IL-6, which explains the higher IL-6 in the fermented soy milk group compared to the unfermented soy milk group. However, in contradiction to the current study on *Streptococcus alactolyticus* and *Streptococcus gallolyticus* [44], there may be an interlocking effect of flora and soy milk.

The present results suggest that broiler intestinal microbiota diversity may be altered after *B. amyloliquefaciens* D1 fermentation soy milk supplementation. *B. amyloliquefaciens* D1 fermented soy milk supplementation resulted in more pronounced microbial aggregation, which was closer in the PCA plot. In a study by Hanifi et al., supplementation with *B. subtilis* did not alter the diversity of the entire microbial community in broilers, presumably inhibiting the growth of some undesirable opportunistic pathogens [45]. In the present study, supplementation with *B. amyloliquefaciens* D1 fermented soy milk caused slight fluctuations in species richness and the diversity of broiler intestinal microbiota but did not appear to be significant. We speculate that *B. amyloliquefaciens* D1 fermented soy milk not only promoted the growth of beneficial microorganisms but also inhibited the growth of opportunistic pathogens. 

The addition of D1 fermented soy milk to diets can modulate host immune function, improve intestinal microbiota, and degrade LPS in vivo. Soy milk is wasted in vast quantities during food manufacturing, yet using D1 fermented soy milk for chicken rearing can be economical and effective. This study suggests that the probiotic effects of the D1 strain could lead to promising uses for this strain in the food and feed industry for reducing inflammation, enhancing nutrition, and enhancing absorption in the body. To confirm the impact of D1 fermented soy milk on intestinal microbiota, it is imperative to expand these investigations to comprehensive, well-designed mouse studies with large sample sizes.

## 5. Conclusions

In conclusion, feeding unfermented soy milk to bearded chickens improved their intestinal microbiota compared to broilers solely fed regular chicken feed. *B. amyloliquefaciens* D1 fermented soymilk increased ADG in bearded chickens. *B. amyloliquefaciens* D1 fermented soy milk significantly reduced the abundance of pathogenic bacteria such as *Streptococcus alactolyticus* and *Streptococcus gallolyticus* in the *Streptococcus* and *Bacteroides fragilis* in *Bacteroides* by increasing the abundance of beneficial bacteria such as *Tidjanibacte*, *Ligilactobacillus*, *Faecalibacterium*, *Bifidobacterium*, *Herbivorax*, and *Mediterraneibacter*, and the microbial community of broiler chickens was shifted to a healthier balance. *B. amyloliquefaciens* D1 fermented soy milk changed inflammatory factors and reduced LPS concentrations in broiler serum. Reduced LPS concentration and decreased inflammatory factors have the potential to alleviate inflammation in broilers. These findings suggest that *B. amyloliquefaciens* D1 fermented soy milk can improve animal growth performance by manipulating intestinal microbial bacteria and reducing inflammation in animals.

## Figures and Tables

**Table 1:** Experimental design.

Groups	Number	Intervention Method	Interventions	Dosage (mL/kg)	Intervention Period (d)
F10	6	Mixing	Fermented soy milk	100	35
F50	6	Mixing	Fermented soy milk	500	35
F100	6	Mixing	Fermented soy milk	1000	35
C10	6	Mixing	Unfermented soy milk	100	35
C50	6	Mixing	Unfermented soy milk	500	35
C100	6	Mixing	Unfermented soy milk	1000	35
CK	6	-	-	-	-

**Table 2:** Effect of fermented soy milk on the performance of bearded chickens (7–42 days old).

Projects	CK	F-10	C-10	F-50	C-50	F-100	C-100
Initial weight (g/bird)	48.29	53.62	52.83	47.95	52.35	54.53	55.33
Average daily feed intake (ADFI)(g/d/bird)	38.72	49.84	51.99	48.69	49.25	44.86	45.73
Average daily gain (ADG) (g/d/bird)	16.18	20.28	19.13	20.53	20.15	19.39	18.32
Average feed/Gain ratio (F/G)	2.39	2.46	2.72	2.37	2.44	2.31	2.50

Note: C control group; F fermented soy milk group; numbers are percentages of soy milk added. The CK group is the normal diet group.

**Table 3:** Effect of *B. amyloliquefaciens* D1 fermented soy milk on physiological and biochemical indicators of broiler serum.

Projects	CK	F-10	C-10	F-50	C-50	F-100	C-100
Total protein (TP) (g/L)	33.33	32.00	34.33	32.67	29.67	32.67	27.67
Albumin (ALB) (g/L)	14.20	13.50	14.43	14.17	11.93	13.63	11.20
Globulin (GLOB) (g/L)	19.13	18.50	19.90	18.50	17.73	19.03	16.47
Albumin/Globulin (A/G)	0.74	0.73	0.73	0.77	0.67	0.72	0.68
Alanine aminotransferase(ALT) (U/L)	-	-	-	-	-	-	-
Aspartate aminotransferase (AST) (U/L)	208.33	182.00	195.67	202.33	180.00	232.00	192.67
ALT/AST							
Triglyceride (TG) (mmol/L)	0.37	0.36	0.31	0.28	0.37	0.36	0.33

Note: C control group; F fermentation solution group; numbers are percentages of soy milk addition; CK is the normal diet group.

**Table 4:** Effect of fermented soymilk on the *alpha* diversity index of broiler cecum contents.

Projects	CK	C10	F10	C50	F50	C100
Chao1 Index	9375.36	7116.31	6358.51	10,185.75	7144.04	7715.71
Richness Index	7937.67	3535.00	5182.33	8232.00	5006.67	4002.67
Shannon Index	9.83	7.35	8.38	9.49	8.76	7.07
Simpson Index	0.050	0.076	0.069	0.055	0.065	0.098
ACE Index	9837.41	8412.53	6901.73	10,976.78	8272.03	8926.87

C is the control group; F is the *B. amyloliquefaciens* D1 fermentation group; numbers are percentages of soy milk addition (%); CK is the normal diet group.

## Data Availability

The sequencing data were deposited in NCBI Sequence Read Archive under the accession number SRP409853 (http://www.ncbi.nlm.nih.gov/Traces/sra) (accessed on 2 December 2022).
